# Whole genome sequence of *Citrus yellow vein clearing virus* CA1 isolate

**DOI:** 10.1186/s13104-023-06443-7

**Published:** 2023-08-10

**Authors:** Yongduo Sun, Raymond Yokomi

**Affiliations:** Department of Agriculture - Agricultural Research Service, United States, San Joaquin Valley Agricultural Sciences Center, Parlier, CA 93648 USA

**Keywords:** *Citrus yellow vein clearing virus*, Genome sequencing, Genome annotation, United States

## Abstract

**Objectives:**

*Citrus yellow vein clearing virus* (CYVCV) is an emerging disease that poses a significant threat to the citrus industry in California. In this study, the viral genomic RNA was isolated from Eureka lemon plants in the greenhouse exhibiting CYVCV symptoms. Subsequently, the corresponding DNA genome amplicon was sequenced and annotated. These efforts expand the genotype database of CYVCV, which aims to enhance detection assays, promote understanding of the virus’s genetics and evolution, and support the management of this disease.

**Data description:**

In this report, we present the complete genome sequence of the CYVCV California isolate (CA1). The genome was found to be 7,530 bp in length, with a G + C content of 51.7%. The 5′ and 3′ termini were determined using 5′ and 3′ termini rapid amplification of cDNA ends (RACE) systems. Furthermore, our analysis revealed the presence of six open reading frames (ORFs) potentially encoding proteins. All sequence data and annotation have been deposited in GenBank under the accession number OR037276.1.

## Objective

*Citrus yellow vein clearing virus* (CYVCV) is a serious emerging quarantine disease that poses a threat to the citrus industry in California and across the United States. The symptoms of yellow vein clearing disease induced by the virus vary depending on citrus varieties and environmental conditions [1, 2]. Lemon (*Citrus limon*) and sour orange (*C. aurantium*) trees are severely affected, while a wide range of other citrus cultivars are susceptible but remain asymptomatic [1, 3]. Infected citrus trees displaying symptoms suffer from growth deficiencies, reduced citrus production, accompanied by yellow vein clearing, adaxial water-soaked appearance of veins, leaf distortion, occasional ringspots, and venial necrosis [1–3].

CYVCV can be transmitted through grafting and mechanical means and is naturally vectored by aphids and whiteflies [1, 4]. The first occurrence of yellow vein clearing disease in citrus was reported in lemons and sour orange from Pakistan in 1988 [5]. Since then, it has been detected in Turkey, India, Iran, and China. The rapid spread of CYVCV in China since 2009 has resulted in serious losses in lemon production [6, 7]. In 2022, the routine multi-pest survey conducted by the California Department of Food and Agriculture (CDFA) identified CYVCV-infected citrus trees in urban properties in the cities of Tulare and Visalia, while no CYVCV have been detected in commercial citrus orchards. The United States Department of Agriculture, Animal and Plant Health Inspection Service (USDA, APHIS) has confirmed positive identifications of the disease. The CDFA currently rates CYVCV as a pest of high concern (Pest rating A) [8].

CYVCV belongs to the *Alphaflexiviridae* virus family, *Mandaraivirus* genus, and is a positive-sense flexuous RNA virus [9]. To investigate potential genomic variations among CYVCV populations in California and other geographical regions, the complete genome sequence of the CYVCV CA1 isolate was amplified and sequenced using state-of-the-art long-read sequencing technology (Plasmidsaurus, Oregon). The assembled complete genome sequence was annotated and deposited in GenBank under the accession number OR037276.1. These genome sequences will be utilized for comparative genomics, contributing to a better understanding of the etiology, relationships, and evolution of CYVCV. Moreover, this genomic data will enhance the efficiency of detection assays for CYVCV in the United States.

## Data description

The citrus buds containing CYVCV isolates was collected from a lemon tree in Tulare, California, and grafted onto Eureka lemon seedlings. The grafted plants were maintained in an air-conditioned quarantine greenhouse at the San Joaquin Valley Agricultural Sciences Center in Parlier, California. Total RNA was isolated from a lemon leaf exhibiting yellow vein clearing symptoms using Trizol Reagent (Thermofisher Scientific, USA). A conserved region in the 5′ end, identified through alignment with other reported CYVCV isolates, was utilized to design a virus-specific 5’ race primer (5’-GGTTAGTGGTATTGCCCTGTT-3’). An oligo(dT) primer was used as the 3’ race-specific primer. The 5’ race and 3’ race polymerase chain reaction (PCR) amplicons were purified and cloned into the pGEM-T easy vector (Promega Corp., Wisconsin). The constructed vectors were further sequenced (Plasmidsaurus, Oregon) to obtain the sequences of the CYVCV 5′ and 3′ termini.

Using the 5′ and 3′ termini sequences, the full genome sequence of the CYVCV CA1 isolate was amplified using the Q5 high-fidelity enzyme (New England Biolabs Inc. Ipswich, Maryland) and virus-specific PCR primers (5′ primer-GAAAAGCAAACATAACCAACACACACCC; 3′ primer-CAGAAAATGGAAACTGAAAGCCTGAATATTT). The resulting 7.5 Kb PCR amplicon was sequenced using the latest long-read sequencing technology from Oxford Nanopore Technologies (ONT, Plasmidsaurus, Oregon). The sequencing process involved constructing an amplification-free long-read sequencing library using the newest v14 library prep chemistry, minimal fragmentation of the linear input DNA in a sequence-independent manner, primer-free sequencing of the library using the highly accurate R10.4.1 flow cells (raw data is > 99% accurate), and re-assembly of the raw reads by aligning them against each other to generate a high-accuracy linear consensus sequence (Data file 1, Table 1).

**Table 1:** Overview of data files/data sets

Label	Name of data file/data set	File types (file extension)	Data repository and identifier (DOI or accession number)
*Data file 1*	*Citrus yellow vein clearing virus isolate CA1, complete genome*	*fasta files (.dcm)*	*Genbank* (https://identifiers.org/nucleotide:OR037276.1*)* [10]

The genome of the CYVCV CA1 isolate was found to be 7,530 bp in length, with an average G + C content of 51.7%. The genome sequence data has been deposited in GenBank under the accession number OR037276.1. The six Open Reading Frames (ORFs) were annotated as follows: ORF1 ranges from 80 bp to 5,029 bp; ORF2 ranges from 5,036 bp to 5,714 bp; ORF3 ranges from 5,691 bp to 6,017 bp; ORF4 ranges from 5,944 bp to 6,126 bp; ORF5 ranges from 6,149 bp to 7,126 bp; ORF6 ranges from 6,826 bp to 7,494 bp. The 5′ and 3′ untranslated regions (UTRs) were also characterized. These data significantly expand the sequence database of CYVCV and are expected to enhance detection assays and provide insights into the evolution of this plant pathogenic virus.

## Limitations

The CYVCV CA1 isolate was collected from a single lemon plant.

## Data Availability

The data described in this Data note can be freely and openly accessed on Genbank under accession number OR037276.1. Please see Table 1 and references [10] for details and links to the data.

## Abbreviations

BP: Base pair
CDFA: California Department of Food and Agriculture
CYVCV: Citrus yellow vein clearing virus
ORF: Open Reading Frame
RACE: Rapid amplification of cDNA ends
PCR: Polymerase chain reaction
USDA, APHIS: United States Department of Agriculture, Animal and Plant Health Inspection Service
UTR: Untranslated region
